# [^18^F]SMBT‐1 PET can visualize the spatial distribution of reactive astrogliosis in patients with neurodegenerative dementias

**DOI:** 10.1002/alz.093700

**Published:** 2025-01-09

**Authors:** Nobuyuki Okamura, Kotaro Hiraoka, Ryota Kobayashi, Naoki Tomita, Aiko Ishiki, Berihu Mesfin, Yingying Wu, Ryuichi Harada, Asuka Kikuchi, Kazuko Takeda, Akio Kikuchi, Katsutoshi Furukawa, Hiroshi Watabe, Shunji Mugikura, Shinobu Kawakatsu, Kenji Ishii, Takashi Nihashi, Takashi Kato, Shozo Furumoto, Manabu Tashiro

**Affiliations:** ^1^ Tohoku Medical and Pharmaceutical University, Sendai Japan; ^2^ CYRIC, Tohoku University, Sendai Japan; ^3^ Yamagata University Hospital, Yamagata Japan; ^4^ Tohoku University Hospital, Sendai Japan; ^5^ Fukushima Medical University, Aizu‐Wakamatsu Japan; ^6^ Tokyo Metropolitan Institute for Geriatrics and Gerontology, Tokyo Japan; ^7^ National Center for Geriatrics and Gerontology, Obu Japan

## Abstract

**Background:**

Reactive astrogliosis refers to functional and morphological changes in astrocytes that occur with neuronal damage in numerous neurological conditions. PET tracers targeting monoamine oxidase B (MAO‐B) are used to visualize reactive astrogliosis in the living brain. [18F]SMBT‐1, a MAO‐B selective PET tracer, was developed by modifying the chemical structure of [18F]THK5351. This study investigates the changes in [18F]SMBT‐1 binding in the brains of patients with Alzheimer's disease (AD) and frontotemporal lobar degeneration (FTLD).

**Method:**

The study involved 85 subjects, including healthy controls (HCs), subjects with mild cognitive impairment (MCI), patients with AD dementia, and FTLD including behavioral variant frontotemporal dementia (bvFTD) and semantic dementia (SD). To compare the regional standardized uptake value ratio (SUVR) values between disease groups, 30‐minute dynamic scans were conducted 60 minutes after administration of [18F]SMBT‐1. The cerebellar white matter was used as a reference region for calculating the SUVR values. PiB PET or flutemetamol PET was performed to confirm the existence of amyloid‐ß pathology in the brain.

**Result:**

[18F]SMBT‐1 binding in the cerebral cortex was elevated in amyloid PET‐positive HC, MCI, and AD patients compared to amyloid PET‐negative subjects. The amount of SMBT‐1 binding in the neocortex correlated with changes in cognitive function of HC and MCI subjects. In addition, a distinct spatial pattern of [18F]SMBT‐1 retention was observed in patients with bvFTD and SD, consistent with areas of brain atrophy in the frontal and temporal lobes.

**Conclusion:**

Distinctive patterns of [18F]SMBT‐1 binding were observed in the neocortex of AD and FTLD, indicating its usefulness for the differential diagnosis of dementia. The study also implies that reactive astrogliosis is present before dementia onset, associated with amyloid‐ß accumulation and cognitive decline.

